# A Novel Artificial Intelligence-Based Mobile Application for Pediatric Weight Estimation

**DOI:** 10.3390/jcm14092873

**Published:** 2025-04-22

**Authors:** Sungwoo Choi, Sangun Nah, Ji Eun Moon, Sangsoo Han

**Affiliations:** 1 Department of Emergency Medicine, Soonchunhyang University Bucheon Hospital, Bucheon 14584, Republic of Korea; csw3613@naver.com (S.C.); potter325@naver.com (S.N.); 2 Department of Biostatistics, Clinical Trial Center, Soonchunhyang University Bucheon Hospital, Bucheon 14584, Republic of Korea; moon6188@schmc.ac.kr

**Keywords:** pediatrics, body weight, deep learning, artificial intelligence

## Abstract

**Background/Objectives**: Pediatric drug dosages are typically weight-based. Length-based weight estimation tools used in emergency situations require full body extension, which may cause measurement errors in restricted positions. In this study, we developed and evaluated a weight prediction application using MoveNet’s human pose estimation and a deep neural network (DNN) regression model. **Methods**: This prospective cross-sectional study was conducted from June 2023 to May 2024 and included pediatric patients aged 1 month to 12 years. Weight estimation accuracy was compared between the Pediatric Artificial Intelligence weight-estimating Camera (PAICam) and the Broselow tape (BT) using mean percentage error (MPE), mean absolute percentage error (MAPE), and root mean square percentage error (RMSPE). The percentages of weight estimations within 10% (PW10) and 20% (PW20) of the actual weights were calculated. Intraclass correlation coefficients (ICCs) were used to evaluate agreement between predicted and actual weights. **Results**: In total, 1335 pediatric participants were analyzed (57.4% boys, 42.6% girls), with an average age of 4 years. The BT and PAICam showed comparable performance, with similar values for MPE (−1.44% vs. 5.29%), MAPE (11.28% vs. 12.41%), and RMSPE (3.09% vs. 3.42%). PW10 and PW20 for the BT and PAICam were also similar (52.6% vs. 51.2% and 79.1% vs. 77.7%). ICC values demonstrated strong agreement between actual and predicted weights for both methods (0.959 vs. 0.955). **Conclusions**: PAICam, utilizing deep learning and human pose estimation technology, demonstrated performance and accuracy comparable to the BT. This suggests its potential as an alternative tool for pediatric weight estimation in emergency settings.

## 1. Introduction

Accurate medication dosing is critical in pediatric treatment. However, studies indicate that dosage errors occur in up to 17.8% of hospitalized pediatric patients [1]. One contributing factor to these errors is that pediatric drug dosages are typically determined based on body weight [2]. Although scales offer accurate weight measurements, pediatric patients often have difficulty cooperating. In emergency situations, this challenge is further complicated by factors such as ongoing resuscitation, altered mental status, or physical limitations caused by trauma [3]. These difficulties can result in significant medication errors during pediatric resuscitation. Reports indicate that such errors occur in approximately 41% of cases, with dosage miscalculations accounting for up to 65% of those incidents [4]. Notably, epinephrine dosage errors in the prehospital setting occur in approximately 60% of cases [1]. Given these risks, ensuring accurate weight estimation is essential for the safe and effective delivery of pediatric medications.

In urgent situations, tools such as the Broselow tape (BT) have been considered the gold standard for accurately determining weight and selecting appropriate medication dosages and equipment [5]. Additionally, various length-based weight estimation methods that account for body habitus have been studied [6,7]. However, despite the use of such tools, dosage errors in pediatric patients remain common. A key limitation is that these tools must be separately carried and readily available [8,9]. Furthermore, length-based tapes have the drawback of being unusable if the patient’s height exceeds the tape’s length [10]. To address these limitations, recent advancements in technology have led to the development of weight estimation methods using applications on smart devices. These applications measure length based on the head-to-heel landmark while the patient lies in a fully extended position to estimate weight [9,10,11,12]. However, there are concerns that measurement errors may occur if the patient’s position is restricted or if cooperation is not easily achieved.

Artificial intelligence (AI) is increasingly transforming the healthcare landscape by enabling data-driven decision-making, enhancing diagnostic accuracy, and optimizing clinical workflows. In particular, AI-based mobile applications have emerged as practical tools in various medical contexts due to their portability, accessibility, and real-time processing capabilities. These applications combine machine learning algorithms with smartphone sensors and imaging capabilities to support disease screening, triage, and clinical decision-support tasks. For example, recent studies have demonstrated the effectiveness of AI-powered mobile platforms in detecting conditions such as Coronavirus disease 2019 [13] and classifying hematological malignancies like B-cell acute lymphoblastic leukemia using lightweight convolutional neural networks (CNNs) [14]. These findings underscore the potential of AI-integrated mobile health solutions to improve access to care, especially in resource-limited settings.

In pediatric emergency care, where rapid and accurate weight estimation is critical for appropriate medication dosing, AI-based mobile solutions may offer a valuable alternative to conventional length-based tools. These applications provide high portability and accessibility, requiring only a smartphone or tablet, and allow for rapid, contactless weight estimation. By integrating human pose estimation algorithms and learning models, AI-based approaches can analyze complex anatomical landmarks and body proportions to generate individualized predictions. Moreover, the learning capacity of AI models enables continuous performance enhancement through real-time data accumulation and model retraining. Such adaptability is advantageous in acute care settings where time-sensitive, accurate weight estimation is critical for safe and effective medication dosing.

In this study, we aimed to address these challenges by utilizing MoveNet, a human pose estimation model [15]. Additionally, we implemented a deep neural network (DNN) regression model AI-based deep learning to minimize errors in weight prediction. Our objective was to evaluate the accuracy and correlation of the predicted weight generated by the application by comparing it with the weight estimated using the BT and the actual weight.

## 2. Materials and Methods

### 2.1. Study Population and Design

This single-blinded, prospective cross-sectional study was approved by our institutional review board (IRB No. 2021-07-005). The study was conducted in the emergency department of a tertiary university hospital in Gyeonggi-do, South Korea, from June 2023 to May 2024. We enrolled pediatric participants aged 1 month to 12 years. Children were excluded if they refused to participate, fell outside the BT height range (<45.9 or >146.5 cm), were uncooperative, or required an emergency procedure [16]. The study process was thoroughly explained to the pediatric participants and their legal guardians, and informed consent was obtained as a requirement.

### 2.2. Pediatric Artificial Intelligence Weight-Estimating Camera (PAICam) and DNN

**Figure A1:**
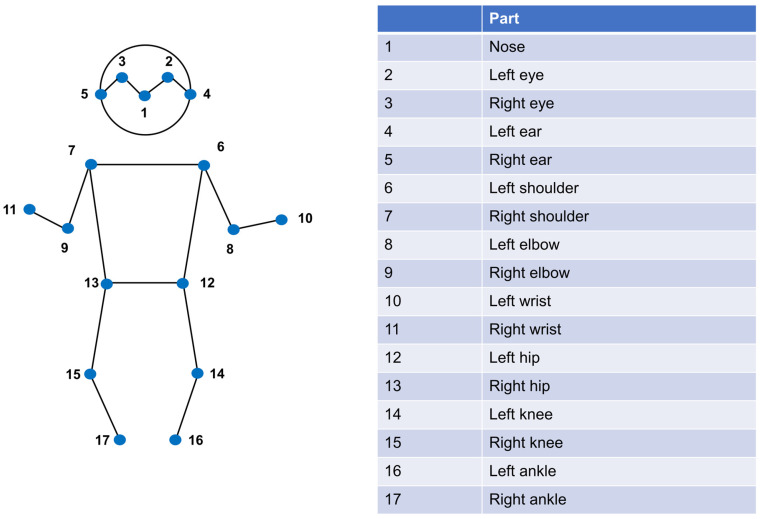
Seventeen joint coordinates were extracted by MoveNet.

**Figure A2:**
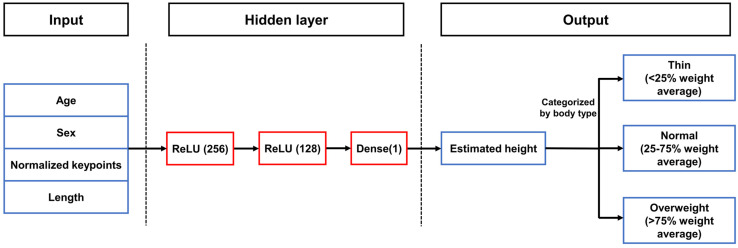
A DNN regression model architecture consisted of three layers.

The PAICam is an iOS mobile application based on deep learning that enables pediatric weight estimation by deriving estimated height from patient photographs. The PAICam was developed by Heartverse Inc. (Seoul, Republic of Korea) using the python and standard deep learning frameworks. The application employs a DNN regression model for AI-based deep learning. Data from 278 pediatric patients under the age of 12 years were used for model training (222 cases for training and 56 cases for validation) following an 80:20 training–validation split. Using MoveNet, 17 joint coordinates (normalized key points) were extracted from pediatric photographs, and the Euclidean distances between each key point were calculated and used as input features (Figure A1). A DNN regression model was constructed using age, sex, and MoveNet-extracted data as inputs. The model architecture consisted of three layers in total, with the first hidden layer containing 256 units and the second hidden layer containing 128 units. Both hidden layers incorporated nonlinearity using the rectified linear unit activation function, while the output layer did not use an activation function (Figure A2). The model’s loss function was mean squared error, and the Adam optimizer was used for optimization. The learning rate was set to 0.001, determined as the most appropriate value through experimentation. To prevent overfitting, the dropout technique was applied with a dropout rate of 0.1.

This specific architecture was selected based on its performance in preliminary testing and its suitability for deployment in a mobile application. Given the limited training dataset and the need for computational efficiency, a simple fully connected feedforward network was favored over more complex models [17,18]. We briefly explored convolutional and residual architectures, but they showed no meaningful performance improvement in our early experiments and increased the risk of overfitting [18]. Therefore, the final architecture was designed to balance accuracy, simplicity, and practical applicability. The model was implemented using TensorFlow 2.0 and Keras, and training was performed over 200 epochs with a batch size of 16. The extracted 17 joint keypoints from MoveNet were normalized, and Euclidean distances were calculated only between anatomically connected keypoint pairs, following the predefined skeletal structure. These distances were then flattened into a one-dimensional feature vector. This feature vector was concatenated with patient metadata, including sex, age, and visually estimated body type (thin, normal, overweight), and served as the input to the DNN. Model weights were initialized using the He normal initializer to support convergence with ReLU activation functions. Early stopping was applied based on validation loss to avoid overfitting. Once training was completed, the model was exported in a mobile-compatible format and integrated into the PAICam iOS application to enable efficient on-device inference without requiring cloud connectivity.

**Figure 1:**
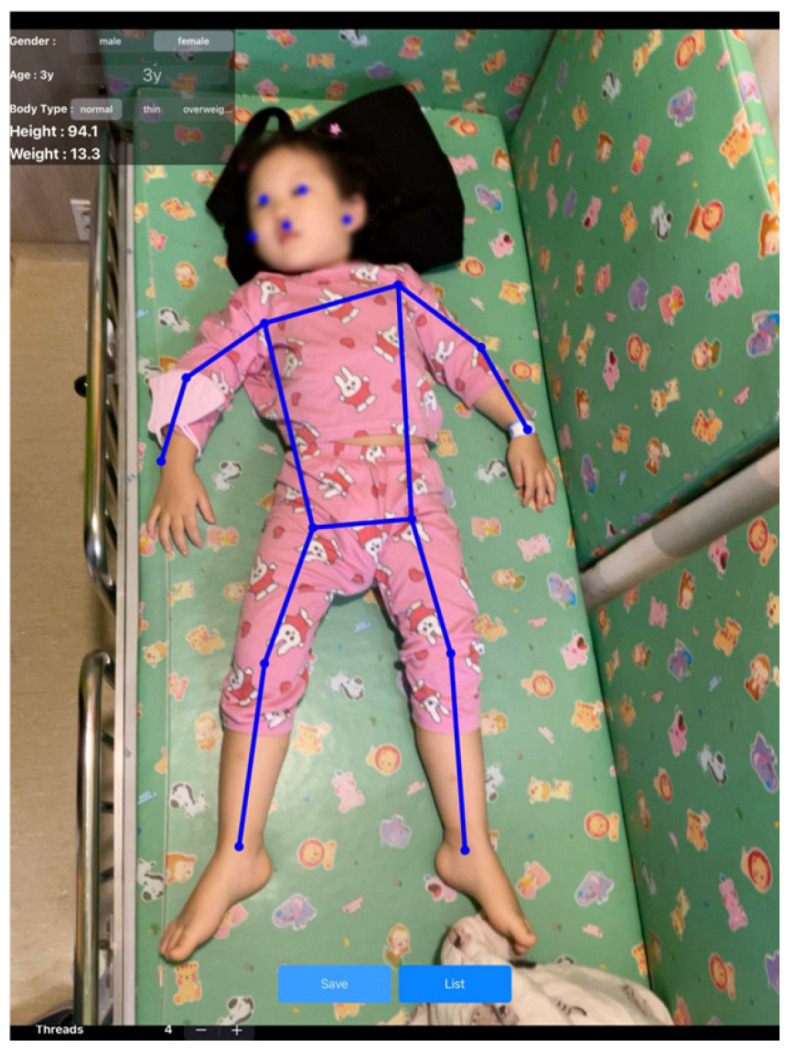
Result screen of PAICam. Key points and connection lines extracted by MoveNet are displayed above the participant. The top left section allows the user to input the participant’s sex, age, and body type, while the final estimated height and weight values are presented.

**Figure A3:**
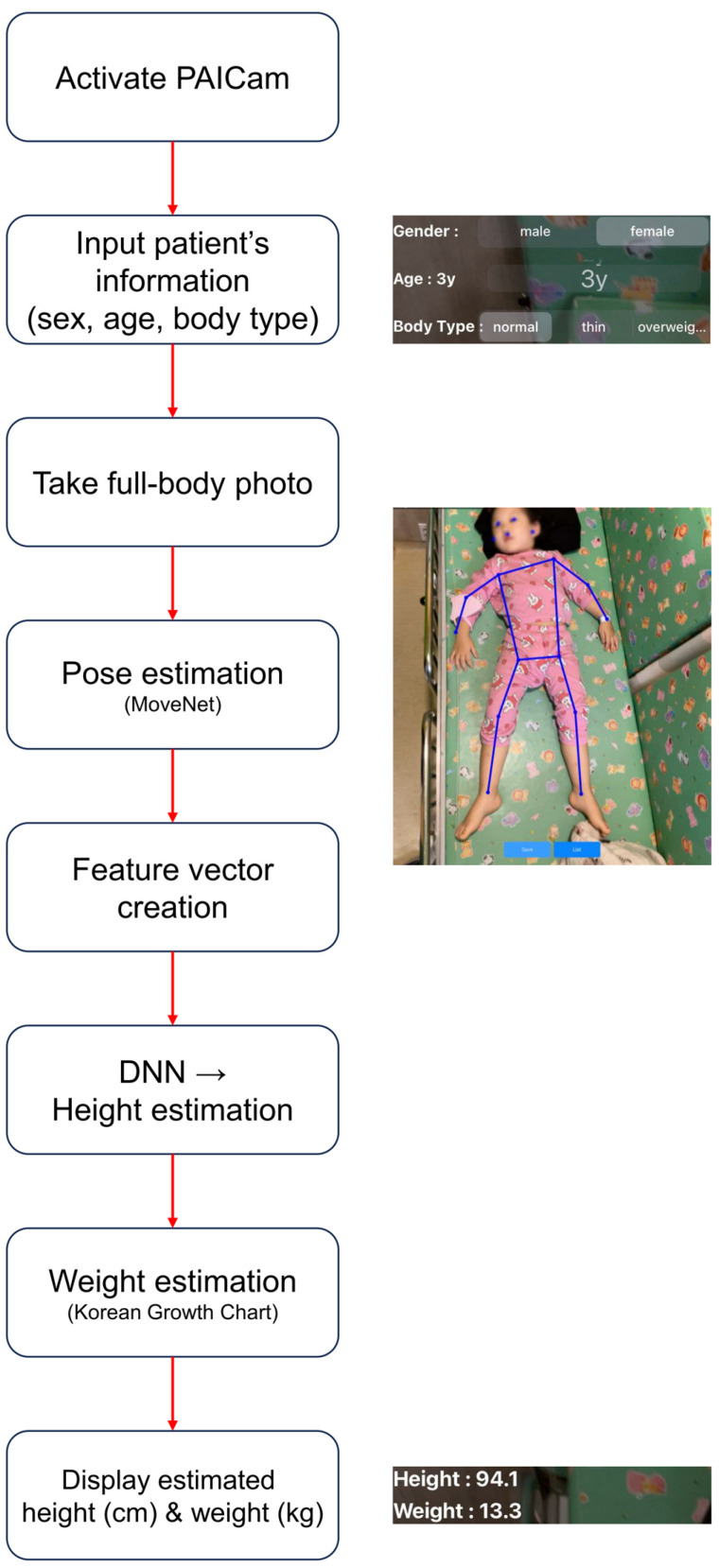
Workflow of the PAICam system for pediatric weight estimation in clinical practice.

The PAICam application features a streamlined and intuitive interface suitable for use in emergency clinical environments. In the upper left section of the main screen, users can input patient’s information—including sex, age, and estimated body type. After entering this information, the user captures a full-body photograph of the patient using the device’s rear-facing camera. The final result screen displays the estimated height (cm) and weight (kg) based on the 2017 Korean National Growth Charts (Figure 1). Estimated weights were categorized according to body type as follows: thin (<25% weight average), normal (25–75% weight average), and overweight (>75% weight average) [7]. A detailed illustration of the PAICam usage workflow in clinical settings is provided in Figure A3.

### 2.3. Data Collection and Sample Size

The participants’ age, sex, and actual height and weight were collected from the electronic medical records. Upon arrival at the emergency department, the actual weight and height were measured by a triage nurse who was blinded to the study’s objective. Weight measurements were conducted to an accuracy of 0.1 kg, while height measurements were accurate to within 0.1 cm. Participants were positioned either on an electronic scale (HM-201; Fanics, Busan, Republic of Korea) if they were able to stand or in a supine position if they were unable to stand, with measurements taken using a measuring device (BF-100A; Fanics). Next, five emergency medical technicians (EMTs) measured the patient’s height using the BT (2017) and PAICam. Prior to data collection, the EMTs had completed a 20-min training session on the utilization of each method and body type determination (thin, normal, and overweight). The participants were asked to lie down fully extended on the bed, and EMTs estimated their weight using both the BT and PAICam. The EMTs captured participant photographs using the PAICam application and entered each participant’s age, sex, and visually estimated body type to obtain the estimated body weight. All photographs were taken using an iPad Pro 12.9 (Apple Inc., Cupertino, CA, USA). The EMTs remained blinded to the actual weights and heights of the participants until all measurements were completed. A sample size of at least 1016 participants was calculated to achieve a power of 80% with a two-sided alpha risk of 0.05. This estimation was based on the assumption that the PAICam would produce a proportion of weights estimated within 10% of actual weight (PW10) that is 10% better than BT, given a reported PW10 of BT at 60%.

### 2.4. Data Analysis

For data analyses, we used R software version 4.2.2 (R Development Core Team, Vienna, Austria). Categorical variables are presented as absolute number with percentage, while continuous variables are expressed as median with interquartile range (IQR). The participants were categorized into three age subgroups for analysis: 1–12 months, 2–5 years, and 6–12 years [19]. The performances of the BT and PAICam were assessed using the mean percentage error (MPE), mean absolute percentage error (MAPE), and root mean square percentage error (RMSPE). The MPE was used to evaluate measurement bias by determining whether the results showed underperformance or overperformance. The MAPE and RMSPE were analyzed to assess overall measurement precision. Additionally, PW10 and PW20 were calculated to evaluate the overall measurement accuracy. Intraclass correlation coefficients (ICCs) were used to compare the predicted and actual weights, and Bland–Altman plots were prepared to visualize agreement between the predicted and actual weights. ICC values were classified as poor (<0.25), low (0.25–0.49), moderate (0.50–0.69), good (0.70–0.89), or excellent (≥0.90) [4]. Differences were considered statistically significant when the *p*-value was <0.05.

## 3. Results

**Figure 2:**
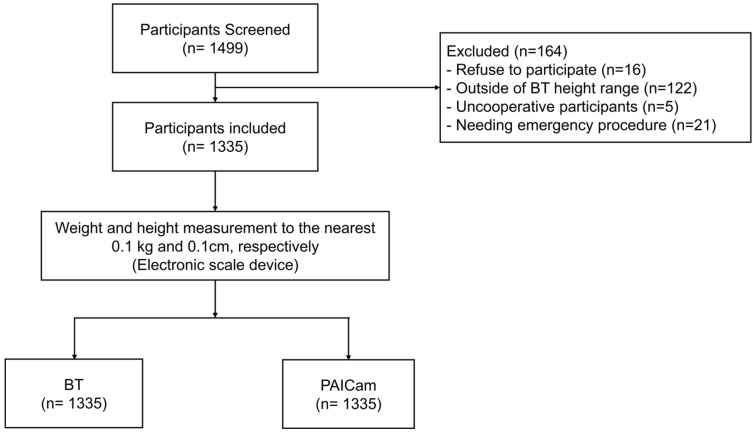
Flow chart of participant selection. Abbreviations: BT, Broselow tape; PAICam, Pediatric Artificial Intelligence weight-estimating Camera.

A total of 1499 pediatric participants were included and analyzed. Of the participants, eight who refused to participate, 122 who were outside the BT height range, five who displayed uncooperative behavior, and 21 who needed emergency procedure were excluded. Consequently, 1335 patients were enrolled in our study (Figure 2).

### 3.1. General Characteristics

In total, 1499 pediatric participants were included in the study. Of these participants, eight refused to participate, 122 were outside the BT height range, five exhibited uncooperative behavior, and 21 required emergency procedures, leading to their exclusion. Consequently, 1335 patients were enrolled and analyzed in our study (Table 1).

### 3.2. Performance of Each Weight Estimation Method

Although the PAICam model was trained on a relatively small dataset (n = 278), it demonstrated comparable performance to the BT when evaluated on a separate clinical dataset consisting of 1335 pediatric patients. The BT had an MPE of −1.44%, MAPE of 11.28%, and RMSPE of 3.09%. The PAICam had an MPE of 5.29%, MAPE of 12.41%, and RMSPE of 3.42%. The limits of agreement were −6.05 to 6.06 for the BT and −5.12 to 7.48 for the PAICam. In the subgroup analysis, the RMSPE values were nearly identical across all subgroups. For all participants, PW10 and PW20 for the BT and PAICam were similar (52.6% vs. 51.2% and 79.1% vs. 77.7%, respectively). The same trend was observed across all subgroups (Table 2).

### 3.3. Correlation Between Actual and Predicted Weight

**Figure 3:**
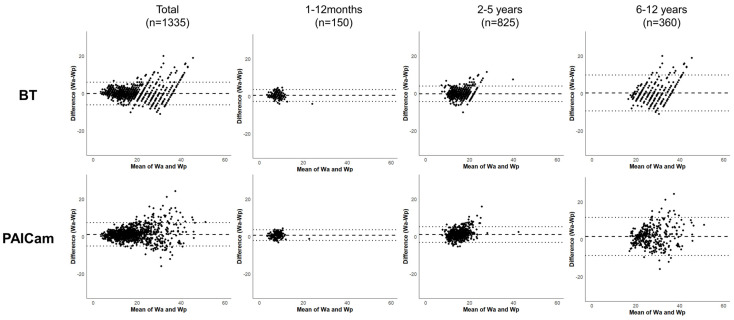
Bland–Altman plots for BT and PAICam with 95% limits of agreement. Abbreviations: BT, Broselow tape; PAICam, Pediatric Artificial Intelligence weight-estimating Camera.

For all participants, the ICC of the BT and PAICam was 0.959 and 0.955, respectively. Additionally, the ICC values for the BT and PAICam were nearly identical across all age subgroups (1–12 months, 0.870 vs. 0.872; 2–5 years, 0.900 vs. 0.889; 6–12 years, 0.829 vs. 0.830) (Table 3). The Bland–Altman plot presents the agreement between actual and predicted weights for both the BT and PAICam across all participants and age subgroups (Figure 3). In the overall dataset, the differences were generally centered around the mean difference line, without noticeable systematic bias or trend across the range of measurements. While some variation was observed among the age subgroups, the distribution of differences remained within similar 95% limits of agreement for both methods.

## 4. Discussion

In this study, we developed and validated a pediatric weight prediction smart device application using a DNN regression model and MoveNet. A key advantage of using a DNN regression model is its ability to learn from large datasets and continuously improve results through real-time learning, enhancing accuracy. This approach is widely applied in the medical field for various applications [20,21,22]. We trained the model with a total of 278 cases, and the results demonstrated that the overall performance and accuracy of the application were comparable to the current gold standard, BT, used in emergency situations. Additionally, the application exhibited an excellent correlation with actual body weight. To address the limitations of previous weight prediction tools, which required patients to be in a supine position, we integrated MoveNet. MoveNet detects posture in real time by identifying 17 joint landmarks and has been shown to outperform other pose detection tools [15]. To the best of our knowledge, no previous applications have utilized AI learning models or human pose estimation tools for pediatric weight prediction via smart devices.

Several studies have explored the use of smart devices for pediatric weight prediction, demonstrating a correlation between the predicted and actual body weight [9,10,11,12]. These applications offer significant advantages over traditional methods, such as length-based tapes, which must be separately carried and prepared. By contrast, smart device applications require only a mobile device, making them far more portable and accessible [9]. This advantage is particularly relevant given the global smartphone penetration rate, which reached 78% in 2020 and is projected to rise to 91% by 2025 [23], further increasing the practicality and accessibility of such applications. However, despite these benefits, certain limitations remain. Previously developed applications often required additional reference points or markings next to the patient, which could be cumbersome [10,12]. Some applications have allowed for direct recognition of the patient, measuring head-to-heel length without additional setup. However, for accuracy, the patient still needed to be in a supine position with full extension [9,11]. In real emergency situations, such as cardiac arrest, seizures, or major trauma, maintaining this position is often impractical. In this regard, the application used in this study, which integrates MoveNet, offers a distinct advantage by recognizing 17 joint landmarks, allowing for weight estimation not only from fixed positions but also from more flexible postures.

When comparing the performance of the BT and PAICam, both demonstrated similar levels of precision (MAPE, RMSPE) and accuracy (PW10, PW20). However, the PAICam showed slightly lower values, which may be attributed to the smaller size of its training dataset. Given the nature of deep learning, better results can be expected as more data are incorporated into training datasets. Additionally, the BT underperformed across all participants, but when analyzed by subgroups, it only showed overperformance in the 1–12-month age group. By contrast, the PAICam overperformed across all participants, displaying a similar pattern in the subgroups. These differences are likely due to the different pediatric growth data each tool used as a reference. The data used to develop BT are based on the National Center for Health Statistics and the National Health and Nutrition Examination Survey [24]. Conversely, the PAICam was designed based on the Korean National Growth Charts, suggesting that regional and ethnic differences in Korean pediatric populations were reflected in the results. In terms of the correlation with actual body weight, the PAICam demonstrated excellent performance across all participants and showed a good correlation when analyzed by subgroup. Considering these factors, the weight prediction using the PAICam in this study demonstrated meaningful performance. However, some errors could potentially be reduced through training with a larger dataset, which may further improve accuracy.

This study has several strengths that underscore its clinical significance. To the best of our knowledge, it is one of the first to integrate deep learning and human pose estimation (MoveNet) for pediatric weight prediction, offering greater flexibility in patient positioning than traditional length-based tools such as the BT. The prospective, single-blinded design and large sample size (1335 participants) enhance the reliability and generalizability of the findings. Additionally, the PAICam provides a highly accessible and portable solution, requiring only a smartphone, making it suitable for both hospital and prehospital settings. Unlike static weight estimation tools, the AI-powered model continuously improves as more data become available, highlighting its potential for even greater accuracy in the future [25].

Despite these strengths, this study has several limitations. First, a direct comparison of body weight prediction with medication dosage and tool size—which are the ultimate goals of accurate weight measurement—was not conducted. Because the primary purpose of weight prediction is to guide medication dosing and equipment selection, future research should focus on incorporating this functionality. Second, although the study sample size was sufficient, the relatively small training dataset may have introduced challenges such as overfitting or reduced reproducibility, potentially leading to unstable performance. However, because the PAICam is based on deep learning technology, its algorithm can improve in real time as more data are input by users [25], which has the potential to significantly enhance accuracy over time. Third, this research was conducted as a single-center study using growth data exclusively from Korean children. Therefore, additional research is needed to evaluate the performance of PAICam in diverse medical settings, including out-of-hospital environments or ambulance use, to determine its broader applicability. Lastly, we were not able to conduct an ablation study to assess the contributions of specific components such as pose estimation or body type classification. This is because these elements were not independently trained or designed to be modular; rather, they were treated as externally generated input features within a fixed processing pipeline based on deep learning outputs. Future work with a more flexible model architecture may allow for such evaluations.

## 5. Conclusions

This prospective cross-sectional study showed that the PAICam, an AI-based mobile application, demonstrated weight estimation accuracy comparable to the BT in pediatric patients. These findings suggest that AI-powered applications could serve as a viable alternative method for pediatric weight estimation in emergency care.

## Figures and Tables

**Table 1:** General characteristics of the study participants.

	Total (N = 1335)	1–12 Months (n = 150)	2–5 Years (n = 825)	6–12 Years (n = 360)
Age, years	4 [2–6]	0.6 [0.3–0.8]	3 [2–4]	7 [6–9]
Sex				
Male	767 (57.4)	77 (51.3)	480 (58.2)	210 (58.3)
Female	568 (42.6)	73 (48.7)	345 (41.8)	150 (41.7)
Weight, kg	16 [12.5–22]	8.6 [7.0–9.7]	15 [12.8–17.2]	26.4 [23.0–32.0]
Height, cm	102 [88–117]	68.8 [32.1–73.5]	97.2 [88.9–105.1]	127.0 [120.0–134.6]
BMI, kg/m^2^	16.1 [15.0–17.8]	17.5 [15.6–19.6]	15.9 [14.9–17.2]	16.3 [15.3–18.6]
Body type				
Thin	58 (4.4)	0 (0.0)	22 (2.7)	36 (10.0)
Normal	1171 (87.7)	141 (94.0)	749 (90.8)	281 (78.1)
Overweight	106 (7.9)	9 (6.0)	54 (6.5)	43 (11.9)

Values are expressed as median [interquartile range] or n (%). Abbreviation: BMI, body mass index.

**Table 2:** Performances of the BT and PAICam.

	BT	PAICam
**All participants (N = 1335)**		
MPE, %	−1.44	5.29
MAPE, %	11.28	12.41
RMSPE, %	3.09	3.42
LOA, kg	−6.05 to 6.06	−5.12 to 7.48
PW10, %	52.6	51.2
PW20, %	79.1	77.7
**1–12 months of age (n = 150)**		
MPE, %	5.08	6.79
MAPE, %	14.55	16.1
RMSPE, %	1.67	1.62
LOA, kg	−2.64 to 3.63	−2.17 to 3.57
PW10, %	43.3	42
PW20, %	68	66.7
**2–5 years of age (n = 825)**		
MPE, %	−2.57	5.74
MAPE, %	10.22	10.91
RMSPE, %	2.14	2.41
LOA, kg	−4.37 to 3.99	−3.09 to 5.31
PW10, %	61.33	60.61
PW20, %	87.88	86.67
**6–12 years of age (n = 360)**		
MPE, %	−1.57	3.65
MAPE, %	12.3	14.3
RMSPE, %	4.87	5.39
LOA, kg	−9.3 to 9.79	−8.58 to 11.69
PW10, %	46.4	43.3
PW20, %	78.6	76.4

Abbreviations: BT, Broselow tape; PAICam, Pediatric Artificial Intelligence weight-estimating Camera; MPE, mean percentage error; MAPE, mean absolute percentage error; RMSPE, root mean square percentage error; LOA, limits of agreement (95% confidence intervals); PW10, percentage of weight estimations within 10% of actual weight; PW20, percentage of weight estimations within 20% of actual weight. Note: measures of precision, accuracy, and bias are classified by age group.

**Table 3:** Correlations between estimated and actual weights in various age subgroups.

	BT			PAICam		
	ICC	95% CI	*p*-Value	ICC	95% CI	*p*-Value
All patients (N = 1335)	0.959	0.955–0.963	<0.001	0.955	0.950–0.960	<0.001
1–12 months (n = 150)	0.870	0.821–0.906	<0.001	0.872	0.823–0.907	<0.001
2–5 years (n = 825)	0.900	0.886–0.913	<0.001	0.889	0.872–0.903	<0.001
6–12 years (n = 360)	0.829	0.790–0.861	<0.001	0.830	0.791–0.862	<0.001

Abbreviations: BT, Broselow tape; PAICam, Pediatric Artificial Intelligence weight-estimating Camera; ICC, intraclass correlation coefficient; CI, confidence interval.

## Data Availability

The data and materials of this study are available from the corresponding author (Sangsoo Han; brayden0819@daum.net) or Institutional Review Board of Soonchunhyang University Bucheon Hospital (20200817@schmc.ac.kr, +82-32-621-6363) upon reasonable request. The application code is currently not publicly available due to proprietary constraints. However, a sample version of the algorithm and anonymized dataset can be shared with academic researchers upon reasonable request to the corresponding author.

## Abbreviations

The following abbreviations are used in this manuscript:

AI	Artificial Intelligence
BT	Broselow Tape
DNN	Deep Neural Network
ICC	Intraclass Correlation Coefficient
IRB	Institutional Review Board
MAPE	Mean Absolute Percentage Error
MPE	Mean Percentage Error
PAICam	Pediatric Artificial Intelligence weight-estimating Camera
PW10	Percentage of Weight Estimations within 10% of Actual Weight
PW20	Percentage of Weight Estimations within 20% of Actual Weight
RMSPE	Root Mean Square Percentage Error
